# Reelin Supplementation Into the Hippocampus Rescues Abnormal Behavior in a Mouse Model of Neurodevelopmental Disorders

**DOI:** 10.3389/fncel.2020.00285

**Published:** 2020-09-02

**Authors:** Daisuke Ibi, Genki Nakasai, Nayu Koide, Masahito Sawahata, Takao Kohno, Rika Takaba, Taku Nagai, Mitsuharu Hattori, Toshitaka Nabeshima, Kiyofumi Yamada, Masayuki Hiramatsu

**Affiliations:** ^1^Department of Chemical Pharmacology, Faculty of Pharmacy, Meijo University, Nagoya, Japan; ^2^Department of Neuropsychopharmacology and Hospital Pharmacy, Nagoya University Graduate School of Medicine, Nagoya, Japan; ^3^Department of Biomedical Science, Graduate School of Pharmaceutical Sciences, Nagoya City University, Nagoya, Japan; ^4^Project Office for Neuropsychological Research Center, Fujita Health University, Toyoake, Japan; ^5^Advanced Diagnostic System Research Laboratory, Fujita Health University, Graduate School of Health Sciences, Toyoake, Japan

**Keywords:** maternal immune activation, Reelin, schizophrenia, autism spectrum disorder, neurodevelopmental disorders

## Abstract

In the majority of schizophrenia patients, chronic atypical antipsychotic administration produces a significant reduction in or even complete remission of psychotic symptoms such as hallucinations and delusions. However, these drugs are not effective in improving cognitive and emotional deficits in patients with schizophrenia. Atypical antipsychotic drugs have a high affinity for the dopamine D_2_ receptor, and a modest affinity for the serotonin 5-HT_2A_ receptor. The cognitive and emotional deficits in schizophrenia are thought to involve neural networks beyond the classical dopaminergic mesolimbic pathway, however, including serotonergic systems. For example, mutations in the *RELN* gene, which encodes Reelin, an extracellular matrix protein involved in neural development and synaptic plasticity, are associated with neurodevelopmental disorders such as schizophrenia and autism spectrum disorder. Furthermore, hippocampal Reelin levels are down-regulated in the brains of both schizophrenic patients and in rodent models of schizophrenia. In the present study, we investigated the effect of Reelin microinjection into the mouse hippocampus on behavioral phenotypes to evaluate the role of Reelin in neurodevelopmental disorders and to test a therapeutic approach that extends beyond classical monoamine targets. To model the cognitive and emotional deficits, as well as histological decreases in Reelin-positive cell numbers and hippocampal synaptoporin distribution, a synaptic vesicle protein, offspring that were prenatally exposed to maternal immune activation were used. Microinjections of recombinant Reelin protein into the hippocampus rescued impairments in object memory and anxiety-like behavior and recruited synaptoporin in the hippocampus in offspring exposed to antenatal inflammation. These results suggest that Reelin supplementation has the potential to treat cognitive and emotional impairments, as well as synaptic disturbances, in patients with neurodevelopmental disorders such as schizophrenia.

## Introduction

Schizophrenia affects up to 1% of the population and can cause life-long disability (Jaaro-Peled et al., 2010). Monoaminergic neurotransmitters are heavily implicated in the pathophysiology of schizophrenia and other psychotic disorders. Atypical antipsychotic drugs have a high affinity for the dopamine D_2_ receptor and serotonin 5-HT_2A_ receptor (Miyamoto et al., 2005) and chronic administration of these drugs can significantly reduce or even completely remit positive psychotic symptoms, such as hallucinations and delusions (Miyamoto et al., 2005; Meltzer, 2013).

Despite some benefits of antipsychotic drugs, they do not often effectively treat the cognitive and emotional symptoms that the majority of schizophrenia patients also have (Nielsen et al., 2015; Howells et al., 2017; Ibi et al., 2017). Cognitive impairment in schizophrenia patients is selective and often includes dysfunction in attention, executive function, and working memory (Rodriguez-Blanco et al., 2017). Emotional symptoms, such as anxiety, are associated with more severe clinical features and worse outcomes (Temmingh and Stein, 2015). Deficits in cognitive processes and difficulties with emotional adjustment account for a significant proportion of psychosocial disabilities in patients with schizophrenia (Millan et al., 2012). Novel therapeutic drugs with strategic targets beyond the classical monoaminergic pathway are required to improve both cognitive deficits and emotional symptoms.

Reelin (*RELN* gene) is a large, secreted extracellular matrix glycoprotein that is a critical player in the modulation of neuronal development, synaptic plasticity, and spine formation/remodeling across the lifespan. During prenatal development, Reelin is expressed and secreted from Cajal-Retzius neurons in the outer layers of the developing cortex. Here, Reelin guides newly born neurons to their correct positions in an inside-out fashion (Frotscher, 2010). During postnatal development, when the Cajal-Retzius cells begin to die out in the cortex and hippocampus (Del Rio et al., 1996) inhibitory GABAergic interneurons begin to express and secrete Reelin (Pesold et al., 1998). This postnatally secreted Reelin acts to modulate axonal and dendritic outgrowth by regulating cytoskeleton stability via multiple independent and interconnected pathways (Wasser and Herz, 2017). In clinical studies, genetic linkage analyses have implicated *RELN* polymorphisms in the pathophysiology of neurodevelopmental diseases such as schizophrenia and autism spectrum disorder (ASD) (Ishii et al., 2016). Postmortem studies have also revealed decreased Reelin expression in the brains and the cerebrospinal fluid of patients with schizophrenia and ASD (Knuesel, 2010; Ishii et al., 2016). These studies suggest that down-regulation of Reelin signaling contributes to the pathophysiology of neurodevelopmental disorders and also raises the possibility that treating Reelin deficiency may help to improve schizophrenia and ASD symptoms (Rogers et al., 2013). Reelin injection into the brain to activate Reelin signaling pathways may thus be a novel therapeutic strategy for the treatment of neurodevelopmental disorders, though this remains untested.

Early disruptions of neurodevelopment contribute to both future psychiatric risk and to the underlying pathophysiology of neurodevelopmental disorders (Kushima et al., 2018; Gumusoglu and Stevens, 2019). There is evidence from multiple clinical studies that demonstrates an association between maternal inflammation during pregnancy and the development of neurodevelopmental disorders in offspring, including schizophrenia (Moreno et al., 2011; Brown, 2012; Kannan and Pletnikov, 2012) and ASD (Patterson, 2011).

Preclinical models of maternal immune activation (MIA) have been developed to examine the physiological mechanisms responsible for this association (Shi et al., 2003; Moreno et al., 2011). One common method of MIA is maternal administration of polyinosinic: polycytidylic acid (poly I:C), which is an agonist of toll-like receptor 3. Poly I:C activates the innate immune system in a manner similar to viral double-stranded RNA, which is produced in viral infection during the genetic replication of single-stranded RNA or as a secondary transcript by DNA viruses (Takeuchi and Akira, 2007).

Behavioral and anatomical differences in the offspring of pregnant mice or rats treated with poly I:C during gestational days (GDs) 9–17 (Shi et al., 2003; Meyer et al., 2005, 2006; Zuckerman and Weiner, 2005; Smith et al., 2007; Holloway et al., 2013), which roughly corresponds to late in the first and early in the second trimester in humans, have been analyzed. This method of MIA increases risk for both schizophrenia and ASD (Ibi and Yamada, 2015). Accumulating evidence demonstrates that the offspring of pregnant mice or rats treated with poly I:C exhibit augmented psychostimulant-induced hyperactivity and impairments in social interaction, prepulse inhibition (PPI), and memory (Meyer et al., 2005; Zuckerman and Weiner, 2005; Smith et al., 2007; Meyer, 2013). These behavioral phenotypes are thought to correspond to cognitive dysfunction and some domains of positive and negative symptoms in patients with schizophrenia (Arguello and Gogos, 2006).

Some characteristic neuropathological features of schizophrenia are also seen in the offspring of mothers treated with polyI:C during pregnancy (Gonzalez-Maeso et al., 2008; Jaaro-Peled et al., 2010). These features include a decrease in the number of Reelin-positive cells in the frontal cortex and hippocampus, reduced dendritic spine density, decreased dopamine D_1_ and metabotropic glutamate 2 receptors, increased 5-HT_2A_ receptors in the prefrontal cortex, loss of parvalbumin (PV) in the hippocampal interneurons, and enhanced tyrosine hydroxylase in the striatum (Holloway et al., 2013; Meyer, 2013; Ibi and Yamada, 2015).

To evaluate the effects of Reelin protein signaling activation in the brain, we injected recombinant Reelin protein into the hippocampi of adult offspring of pregnant C57BL/6J mice that were intraperitoneally treated with poly I:C on GD 9. To the best of our knowledge, this is the first experimental study to investigate the effects of Reelin signaling activation as a therapeutic strategy for cognitive and emotional deficits and synaptic disturbances in an MIA-induced preclinical model of neurodevelopmental disorders.

## Materials and Methods

### Animals

Pregnant C57BL/6J mice at GD 6–7 were obtained from Japan SLC Inc. (Hamamatsu, Japan). Dams were housed individually and habituated to the testing facility for a few days before experimental use. Mice were kept in a regulated environment (24 ± 1°C, 55 ± 5% humidity) under a 12-h light/dark cycle (lights on 7:45 a.m.) and provided food and tap water *ad libitum*. All experimental protocols including the use of laboratory animals were approved by the Animal Ethics Board of Meijo University and followed the guidelines of the Japanese Pharmacological Society (Folia Pharmacol. Japan, 1992, 99: 35A); the Interministerial Decree of May 25th, 1987 (Ministry of Education, Japan); and the National Institutes of Health Guide for the Care and Use of Laboratory Animals (NIH Publications No. 8023, revised 1978). All efforts were made to minimize animal suffering and to reduce the number of animals used.

### Maternal Immune Activation (MIA) Model

At GD 9, pregnant mice received intraperitoneal injections of poly I:C (20 mg/kg; Sigma-Aldrich, St. Louis, MO) (i.p.), as previously (Esslinger et al., 2016; Gonzalez-Liencres et al., 2016). Control mice received intraperitoneal injections of sterile saline (0.9%). After treatment, pregnant mice were singly housed until parturition. Offspring were weaned and sexed on postnatal day (PD) 28. Males and females were caged separately, and littermates of the same sex were housed in groups of 3–6 animals per cage. Both experimental groups (vehicle and poly I:C) were composed of multiple independent litters to prevent litter effects. All litters included offspring of both sexes.

### Purification of Full-Length Reelin

Recombinant Reelin protein was prepared using the Expi293 Expression System (Thermo Fisher Scientific, MA). Expi293F cells were transfected with a full-length mouse Reelin protein, which was fused at the N-terminal with a PA tag (Fujii et al., 2014). After 4 days, the conditioned medium containing the recombinant Reelin was harvested. To purify recombinant Reelin, the conditioned medium was incubated with anti-PA tag antibody (Wako, Osaka, Japan) coated-beads at 4°C overnight. Purified Reelin was then concentrated approximately 10-fold by an Amicon Ultra centrifuge filter (100,000 molecular weight cut-off, Merck, Darmstadt, Germany). To estimate the concentration, Reelin was detected by Coomassie blue staining and compared to bovine serum albumin (Ishii et al., 2015).

### Stereotactic Hippocampal Reelin Injections

Offspring were anesthetized with a mixture (i.p.) of medetomidine hydrochloride (0.3 mg/kg; Wako), midazolam (4 mg/kg; Wako), and butorphanol tartrate (5 mg/kg; Wako), and then positioned between the ear bars of a stereotaxic frame (SR-6N; Narishige, Tokyo, Japan). Full-length Reelin (0.2 pmol/0.5 μL) protein or PBS (control) was delivered bilaterally with a Hamilton syringe at a rate of 0.1 μL/min for a total volume of 0.5 μL on each side. The needle was left in place for 5 min. Injection volume and concentration of recombinant Reelin protein was determined using previous studies (Rogers et al., 2011, 2013; Ishii et al., 2015). The following coordinates were used: -1.75 mm rostrocaudal, -2.0 mm dorsoventral, ±1.0 mm mediolateral from bregma (relative to dura). Immediately after removal of the needle, the skin was closed with tissue adhesive (Vetbond, 3M, St. Paul, MN). The injection point was confirmed by methylene blue microinjection (Supplementary Figure S1).

### Behavioral Analyses

Behavioral analyses in the offspring of both saline- and poly I:C-treated dams were conducted at 8–20 weeks of age. To test for a rescue of the cognitive and emotional deficits seen in the MIA model of schizophrenia by recombinant Reelin injection into the hippocampus, we used a novel object recognition test on Day 4 post Reelin microinjection (Day 0). We then used the open-field test on Day 10 post microinjection.

#### Novel Object Recognition Test

Novel object recognition, a spontaneous form of memory, is derived from curiosity about novel objects (Leger et al., 2013). A novel object recognition test was performed, as described previously (Ibi et al., 2017). Male and female mice were individually habituated to an open box (30 × 30 × 35 cm high) for 3 days. During training sessions, two novel objects were placed in the open field and animals were allowed to explore the objects for 10 min under moderate light (20 lux).

During retention sessions, animals were placed into the same box 24 hr after the training session. One of the familiar objects used during the training sessions was replaced by a novel object, and the mice were allowed to explore the box freely for 5 min. A preference index for the retention session (the ratio of time spent exploring the novel object over the total time spent exploring both objects) was calculated to measure novel object recognition. During the training session, a preference index was also calculated as the ratio of time spent exploring the object that would be replaced by a novel object in the retention session to the total exploration time. The time spent exploring each object in both sessions was also recorded on video for subsequent blind scoring.

#### Open-Field Test

The open-field test poses a conflict between the endogenous mouse exploratory drive and their aversion to exposure in open, illuminated open areas. This test was used to examine both anxiety-like and locomotor behavior (Ibi et al., 2009). Mice were placed in the center of a square open arena (50 × 50 cm, wall height: 35 cm) and allowed to explore it for 60 min under bright illumination conditions (80 lux). Mouse activity was measured automatically using the EthoVision automated tracking program (Noldus Information Technology, Sterling, VA) (Ibi et al., 2009; Udagawa et al., 2015). The open-field was further divided into an inner square (40 × 40 cm) and an outer area (50 × 50 cm), which surrounded the inner square. Mouse movement was measured via a camera mounted directly above the open-field. Measurements included distance and time spent in the inner and outer sections.

#### Spontaneous Locomotor Activity

Each mouse was placed in a standard transparent rectangular rodent cage (25 × 30 × 18 cm) under moderate illumination conditions (15 lux). Locomotor activity was measured for 120 min using an automated system of digital counters with infrared sensors (Scanet SV-10; Melquest Co., Ltd., Japan).

### Immunohistochemistry

Mice were deeply anesthetized with ethyl carbamate (1.5 g/kg i.p., Katayama Chemical, Osaka, Japan) and perfused transcardially with saline, followed by 4% paraformaldehyde in 0.1 M phosphate-buffered saline (PBS, pH 7.4). Mouse brains were removed, post-fixed in the same fixative, and then cryoprotected. Twenty micrometer-thick coronal free-floating brain sections were made using a cryostat. Free-floating sections were transferred to 24-well dishes containing PBS. After blocking with 10% goat serum/PBS for 60 min, mouse anti-synaptophysin (Sigma-Aldrich SAB4200544, 1:500), rabbit anti-synaptoporin (Synaptic Systems, Göttingen, Germany 102002, 1:500), mouse anti-Reelin (Merck-Millipore MAB5364, 1:1000), mouse anti-PV (Sigma-Aldrich P3088, 1:2000), and rat anti-somatostatin (SST) antibodies (Merck-Millipore MAB354, 1:350) diluted in 10% goat serum/PBS were applied to the sections, which were then incubated overnight at 4°C. After washing in PBS, goat anti-mouse Alexa Fluor 568 and anti-rabbit Alexa Fluor 488 antibodies (1:3000; Invitrogen, Eugene, OR) were added to the sections for 2 hr at room temperature. Samples were imaged using an all-in-one Fluorescence Microscope (BZ-700, Keyence, Osaka, Japan) and a confocal-laser scanning microscope (LSM 800; Zeiss, Jene, Germany).

For the quantification of synaptophysin immunoreactivity, mean immunoreactivity signal intensity (Bregma -1.70 to -2.18 mm) in the hippocampus was measured using NIH image 1.62 software (Guan et al., 2009). For the quantification of synaptoporin-immunostained area, the area of immunoreactive region in the hippocampal DG (Bregma -1.70 to -2.18 mm) was measured using Keyence BZ-X Analyzer software (Keyence) according to previous reports (Latchney et al., 2014; Zuko et al., 2016). For quantification of the number of Reelin-, PV- and SST-positive cells, the number of hippocampal immunoreactive cells (Bregma -1.70 to -2.18 mm) in both hemispheres was counted. These analyses were performed by a blinded experimenter.

### Statistical Analyses

Statistical analyses were performed and figures were produced using Prism software version 6 (GraphPad Software, Inc., San Diego, CA). As it was not possible to assume that behavioral data had a Gaussian distribution, these data are expressed as medians and interquartile ranges. Group-wise differences were evaluated using the Mann-Whitney *U*-test for comparisons between two groups.

Quantitative values obtained from immunohistochemistry are expressed as means ± standard errors (SEs). Unpaired *t*-tests were used to compare two groups.

Two-way ANOVAs followed by Tukey-Kramer *post hoc* test was used for multiple comparisons where relevant (e.g., Reelin injection and maternal poly I:C treatment). The percentage of aborted pregnancies was evaluated using a chi-square (x^2^) test. The criterion for statistical significance was *p* < 0.05.

## Results

### General Appearance of MIA Offspring and Conditions of MIA Pregnancy

After acute administration of 20 mg/kg polyI:C (i.p.) at GD 9, pregnant C57BL6J mice had a significantly increased percentage of aborted pregnancies [vehicle 25% (2/8), poly I:C 86% (43/50); x^2^ = 14.76, *p* < 0.001], including either fetal resorptions (abdominal collapse without abortion of the dead fetuses) or frank abortions (premature discharge of dead fetuses). This result was consistent with a previous study (Ehninger et al., 2012). MIA did not affect adult offspring body weight at 9–12 weeks of age (mean ± SE: vehicle 23.53 ± 0.68 g, poly I:C 23.65 ± 0.34 g, *p* = 0.88). Moreover, offspring with MIA were viable and did not display any gross histological abnormalities in the hippocampus (data not shown). These results suggest that MIA negatively affected birth rate, but not offspring growth or the gross structure of offspring hippocampal layers.

### Hippocampal Reelin Expression in MIA Offspring

**FIGURE 1:**
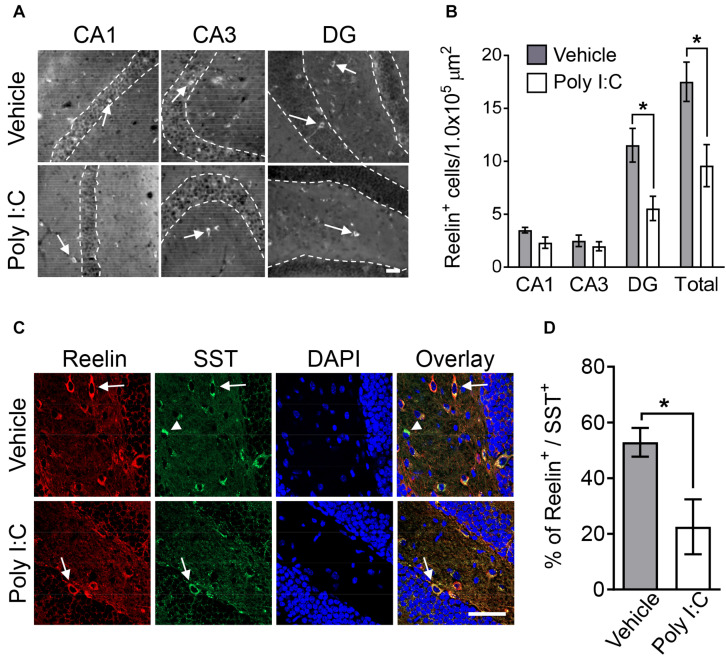
Hippocampal Reelin expression in MIA offspring. Representative photographs showing Reelin-positive cells (arrows) in hippocampal subregions **(A)**. Quantification of Reelin-positive neuron numbers in hippocampal subregions **(B)**. Representative photographs of double-immunostained Reelin- and SST-positive cells (arrows: Reelin-positive/SST-positive, arrowhead: Reelin-negative/SST-positive) in offspring hippocampi **(C)**. Percentage of Reelin-positive cells among SST-positive cells in offspring hippocampi **(D)**. Values are means ± SE [Reelin staining *n* = 6–9 (vehicle: 5 males and a female, poly I:C: 7 males and 2 females), Reelin and SOM double-staining *n* = 3 (each group: 2 males and a female); from three independent dams]. Two-tailed *t*-test comparing MIA offspring vs. vehicle-treated control group offspring **(B,D)**. *p < 0.05. Scale bar: 50 μm.

Given a previous study demonstrating that MIA decreases the number of Reelin-positive cells in the hippocampus (Meyer et al., 2006), we examined Reelin expression in the hippocampi of adult offspring here. MIA decreased the number of Reelin-positive cells in the DG of the adult hippocampus but not in the CA1 or CA3 regions (Figures 1A,B). There was also no difference in the number of Reelin-positive cells in the medial prefrontal cortex between control and MIA offspring (mean ± SE: vehicle 8.41 ± 1.06, poly I:C 7.63 ± 1.40, *p* = 0.65) despite the fact that Reelin is a key modulator of cortical development and lamination (Herz and Chen, 2006).

Dysregulation of inhibitory GABAergic interneuron populations has been implicated in psychiatric disorders, such as schizophrenia, anxiety disorders, and ASD (Tremblay et al., 2016). GABAergic interneurons are classified as PV-, SST-, and serotonin 5-HT_3A_ receptor-positive interneurons. SST-positive neuronal population consist exclusively of Reelin-positive cells (Tremblay et al., 2016). We thus investigated the co-localization of Reelin and SST in the hippocampal DG of MIA offspring. We found that the percentage of Reelin-positive cells among SST-positive interneurons in MIA offspring was smaller than the percentage in controls (Figures 1C,D). Furthermore, the number of Reelin/SST double-positive cells (mean ± SE: vehicle 8.18 ± 0.88, poly I:C 4.26 ± 0.67, *p* < 0.001) and SST-positive cells (means ± SE: vehicle 16.36 ± 1.78, poly I:C 11.13 ± 0.80, *p* < 0.01) in the hippocampal DG was also significantly decreased by MIA.

Previous research has implicated deficits in PV-positive GABAergic interneurons in the pathogenesis of schizophrenia (Jaaro-Peled et al., 2010). Thus, we investigated the number of hippocampal PV-positive interneurons in MIA offspring here. Offspring hippocampal PV-positive interneuron numbers were not changed by MIA (mean ± SE; CA1 region: vehicle 4.06 ± 0.34, poly I:C 3.40 ± 0.72, *p* = 0.35; CA3 region: vehicle 3.86 ± 0.53, poly I:C 2.45 ± 0.62, *p* = 0.11; DG: vehicle 1.85 ± 0.29, poly I:C 2.55 ± 0.35, *p* = 0.14). Together, these results demonstrate that MIA reduced both Reelin- and SST-positive interneurons, but did not change the number of PV-positive neurons.

### Hippocampal Synaptic Proteins in MIA Offspring

Multiple lines of evidence implicate synaptic dysfunction in the hippocampus in schizophrenia symptomatology (Tamminga et al., 2012; Osimo et al., 2018). For example, decreased hippocampal synaptic protein and mRNA levels in patients with schizophrenia have been previously reported (Osimo et al., 2018). Thus, we examined levels of hippocampal synaptic markers in offspring with MIA in the present study.

**FIGURE 2:**
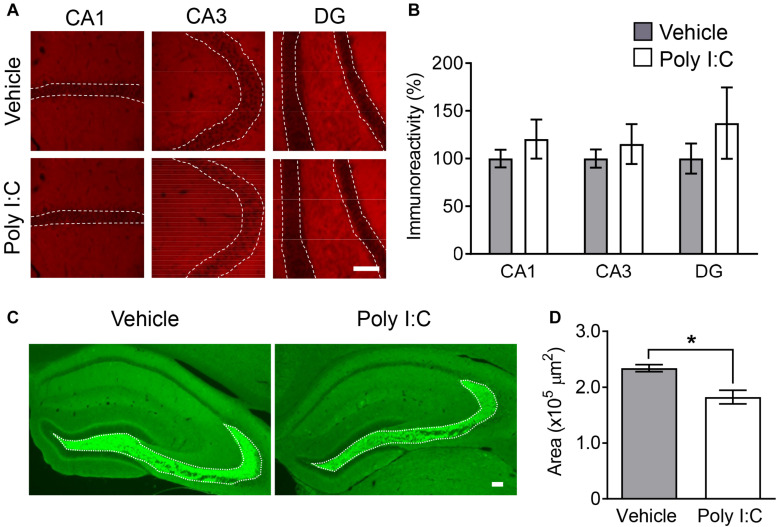
Hippocampal synaptic markers in MIA offspring. Representative photographs showing synaptophysin **(A)** and synaptoporin **(C)** immunoreactivity in offspring hippocampi. Quantification of offspring hippocampal synaptophysin **(B)** and synaptoporin **(D)** immunoreactivity. Values are means ± SE [synaptophysin staining *n* = 4 (each group: 4 males), synaptoporin staining (each group: 2 males and a female); from three independent dams)]. Two-tailed *t*-test comparing MIA offspring vs. vehicle-treated control group offspring **(B,D)**. *p < 0.05. Scale bar: 100 μm.

Immunoreactivity to synaptophysin (also known as synaptophysin 1), a presynaptic terminal marker of active synapses (Guan et al., 2009), was not changed in any hippocampal subregion by MIA (Figures 2A,B). However, immunoreactive area size for synaptoporin, a marker enriched in mossy fiber tracts (also known as synaptophysin 2) (Romer et al., 2011), was significantly decreased in MIA offspring compared to control mice (Figures 2C,D). These findings agree with previous reports of decreased mossy fiber synapse numbers in the hippocampus in schizophrenics (Kolomeets et al., 2007).

### Effect of Reelin Injection on Behavioral Abnormalities in MIA Offspring

**FIGURE 3:**
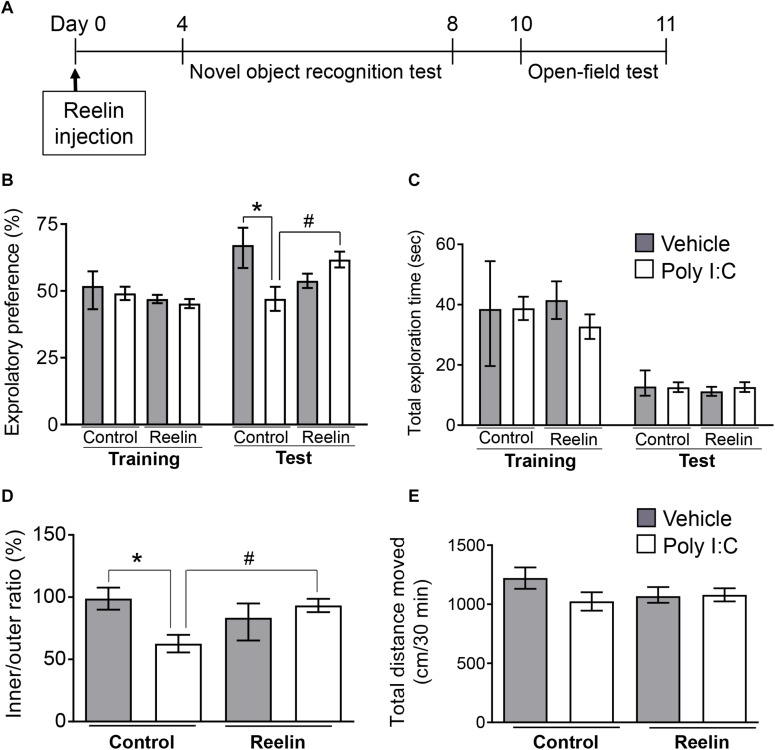
Effect of Reelin injection into the hippocampus on the abnormal behavior of MIA offspring. Experimental schedule for Reelin injection into the hippocampus and subsequent assays **(A)**. Exploratory preference **(B)** and total exploration time **(C)** on the novel object recognition test. The test session was carried out 24 h after the training session. The time spent in the inside/outside (inner/outer ratio) of the field **(D)** and total distance traveled **(E)** in the open-field test, in which mice were allowed to explore the open field freely for 60 min. Exploratory behaviors were analyzed only in the final 30 min of the task due to consistent hyperactivity during the first 30 min. Values represent medians and interquartile ranges [novel object recognition test *n* = 8–12 (control-vehicle: 4 males and 4 females, control-poly I:C: 5 males and 5 females, Reelin-vehicle: 4 males and 4 females, reelin-poly I:C: 6 males and 6 females), open-field test *n* = 8–12 (control-vehicle: 4 males and 4 females, control-poly I:C: 6 males and 6 females, reelin-vehicle: 5 males and 5 females, reelin-poly I:C: 6 males and 6 females); from three or more independent dams]. Tukey-Kramer test *p < 0.05 in the comparison between MIA offspring with hippocampal Reelin injection vs. vehicle-treated control group, ^#^*p* < 0.05 for MIA offspring with hippocampal Reelin vs. PBS treatment.

Given that hippocampal Reelin plays a crucial role in adult neurogenesis, synaptic plasticity and granule cell malformation (Frotscher, 2010; Jakob et al., 2017), a decrease in the number of Reelin-positive cells (Figure 1) may affect hippocampal function. Therefore, we examined the effects of increased exogenous intra-hippocampal Reelin on behavior and hippocampal mossy fiber synapse numbers in MIA offspring. The novel object recognition test was carried out 4 days after Reelin injections and followed by the open-field test on Day 10 post-injection (Figure 3A). This schedule is based on previous studies in which behavioral assays were performed 5–10 days after Reelin microinjection into the lateral ventricle (Rogers et al., 2011, 2013; Ishii et al., 2015).

During novel object recognition task training sessions, all groups exhibited nearly equal exploratory preference for each object (Figure 3B: F_Poly  I:C (1,34)_ = 0.704, *p* = 0.41; F_Reelin (1,34)_ = 3.32, *p* = 0.078; F_Interaction (1,34)_ = 0.0028, *p* = 0.96), indicating that neither MIA nor Reelin injection itself biased object preferences. In the test session, which was carried out 24 hr after the training session, two-way ANOVA showed a significant interaction effect between maternal poly I:C treatment and recombinant Reelin injection (F_Poly  I:C (1,34)_ = 1.576, *p* = 0.22; F_Reelin (1,34)_ = 0.30, *p* = 0.59; F_Interaction (1,34)_ = 11.45, *p* = 0.0018) and *post hoc* analysis revealed that MIA-exposed offspring exhibited significantly lower exploratory preference for the novel object than did control mice (Figure 3B). This suggests that MIA impairs object recognition in offspring.

Deficits in object recognition were rescued with hippocampal Reelin injection, as these mice had significantly higher exploratory preference to the novel object than those of MIA-exposed offspring without Reelin supplementation. Finally, there was no difference in total object exploration time between the training and test sessions among all groups (Figure 3C: Training session F_Poly  I:C (1,34)_ = 0.11, *p* = 0.74; F_Reelin (1,34)_ = 1.042, *p* = 0.31; F_Interaction (1,34)_ = 1.27, *p* = 0.27, Test session F_Poly  I:C (1,34)_ = 0.56, *p* = 0.46; F_Reelin (1,34)_ = 0.16, *p* = 0.69; F_Interaction (1,34)_ = 0.33, *p* = 0.57), suggesting that neither MIA nor Reelin injection affected exploratory behaviors and/or motor function. Taken together, these results demonstrate that MIA impaired object recognition in offspring, which was rescued by hippocampal Reelin injection.

In the open-field test, mice were allowed to explore the open field freely for 60 min, as previously (Audero et al., 2013). Since hyperactivity was observed in MIA offspring during the first 30 min in a novel environment, their exploratory behavior was analyzed only in the final 30 min (Supplementary Figure S2: F_Poly  I:C (1,126)_ = 15.42, *p* = 0.0001; F_*Time (5,126)*_ = 5.30, *p* = 0.0002; F_Interaction (5,126)_ = 0.72, *p* = 0.61). Two-way ANOVA showed a significant interaction effect between maternal poly I:C treatment and recombinant Reelin injection (F_Poly  I:C (1,36)_ = 2.71, *p* = 0.11; F_Reelin (1,36)_ = 0.73, *p* = 0.40; F_Interaction (1,36)_ = 12.45, *p* = 0.0012) and *post hoc* analysis revealed that MIA offspring exhibited a significantly lower ratio of time spent in the inner area divided by that in the outer area (inner/outer ratio) than vehicle-treated control offspring. However, hippocampal Reelin treatment significantly increased ratio of time spent in the inner to outer zones of the open field among MIA-exposed offspring (Figure 3D). There was no difference in the total distance traveled among all groups (Figure 3E: F_Poly  I:C (1,36)_ = 2.12, *p* = 0.15; F_Reelin (1,36)_ = 0.29, *p* = 0.59; F_Interaction (1,36)_ = 1.77, *p* = 0.19), suggesting that neither MIA nor hippocampal Reelin treatment affected exploratory behaviors or motor function in the open-field test. These results demonstrate that MIA increased anxiety-like behaviors in offspring, which were suppressed by hippocampal Reelin injection.

### Effect of Reelin Injection on Synaptoporin Levels in MIA Offspring

**FIGURE 4:**
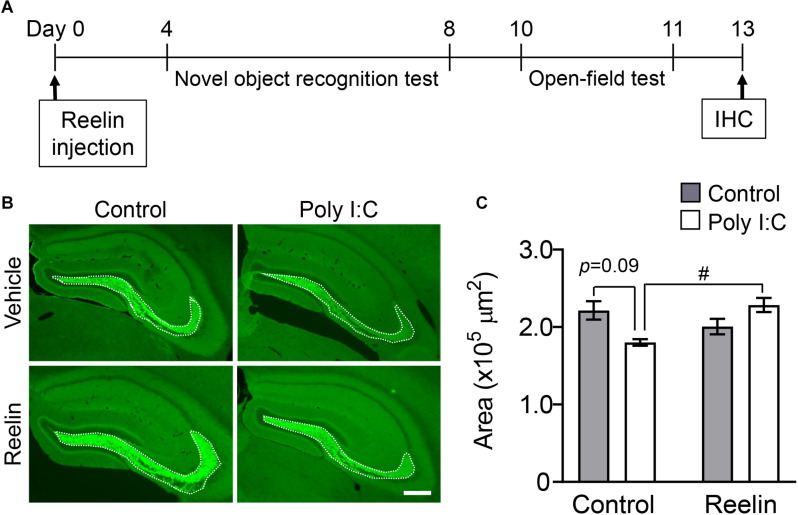
Effect of Reelin injection into the hippocampus on synaptoporin levels in MIA offspring. Experimental schedule of Reelin injection into the hippocampus and subsequent assays **(A)**. Representative photographs showing synaptoporin immunoreactivity in the hippocampi of MIA offspring **(B)**. Quantification of hippocampal synaptoporin immunoreactivity in offspring **(C)**. Values indicate means ± SE [*n* = 3–6 (control-vehicle: 3 males and a female, control-poly I:C: 3 males, reelin-vehicle: 3 males and 2 females, reelin-poly I:C: 3 males and 3 females); from three independent dams]. Tukey-Kramer test ^#^*p* < 0.05 in the comparison between MIA offspring with hippocampal Reelin injection vs. PBS treatment. Scale bar: 300 μm. IHC: Immunohistochemistry.

Thirteen days post-surgery, we tested whether Reelin injection into the hippocampus reversed the down-regulation of synaptoporin-immunostained area in MIA offspring (Figure 4A). Two-way ANOVA showed a significant interaction effect between maternal poly I:C treatment and recombinant Reelin injection (Figure 4C: F_Poly  I:C (1,14)_ = 0.42, *p* = 0.53; F_Reelin (1,14)_ = 1.75, p = 0.21; F_Inteaction (1,14)_ = 11.19, *p* < 0.01) and *post hoc* analysis revealed that the hippocampal synaptoporin-immunopositive area tended to decrease in offspring with MIA compare to controls (Figure 4B), which is consistent with Figures 2C,D. Meanwhile, the decrease of immunoreactive area to synaptoporin in offspring with MIA was significantly reversed by Reelin injection into the hippocampus (Figure 4C), demonstrating that Reelin supplementation may reverse MIA-induced mossy fiber deficits.

## Discussion

In the 65 years since the first antipsychotic was discovered, inhibition of neurotransmission via the dopamine receptor has proven to be an important strategy for the treatment of schizophrenia (Miyamoto et al., 2005; Stroup et al., 2006). However, these therapies are only effective in two-thirds of all patients, and their efficacy is limited by the lack of an effect on negative and cognitive symptoms (Nielsen et al., 2015; Howells et al., 2017). Therefore, there is an urgent need to identify new molecular targets to address the various dimensions of schizophrenia symptoms. In the present study, Reelin injection into the hippocampus reversed impairments in object recognition memory and anxiety-like behavior, as well as the decreased in synaptoporin-immunoreactive area in the hippocampus, in an MIA animal model of schizophrenia. A previous study reported similar effects of Reelin, with intraventricular injections of Reelin rescuing synaptic dysfunction and hippocampus-dependent cognitive impairments in a mouse model of Angelman syndrome, a genetic disorder which impairs nervous system development (Hethorn et al., 2015). These results indicate that Reelin may be a new molecular target for the treatment of neurodevelopmental disorders.

It is critical that synaptic disturbances are assessed to better understand the pathophysiology of neurodevelopmental disorders such as ASD and schizophrenia (Pocklington et al., 2014). Disruptions in synaptic formation and remodeling have been observed in the brains of patients with neurodevelopmental disorders (Penzes et al., 2011). In patients with schizophrenia, decreased protein and/or mRNA levels of various synaptic markers (Osimo et al., 2018) have been reported. These changes in expression levels may serve as a marker of synaptic disturbance. In particular, synaptoporin, which is exclusively enriched in granule cell axons, is a synaptic vesicle marker for hippocampal mossy fibers (Romer et al., 2011). Patients with schizophrenia have both decreased synaptoporin levels (Guillozet-Bongaarts et al., 2014; Chang et al., 2017) and mossy fiber deficits (Kolomeets et al., 2007), indicating that synaptoporin expression may be a good marker for hippocampal synaptic disturbances in schizophrenia. In the presents study, we demonstrated synaptoporin deficit in MIA offspring, even in the absence of gross anatomical alterations in the brain or neuronal loss. Our findings support the hypothesis that neurodevelopmental disturbances, but not neurodegeneration, are central to the etiopathogenesis and disease progression of neurodevelopmental disorders.

MIA leads to hippocampal synapse disturbances, which may be rescued by the activation of the Reelin signaling pathway. In double knockout *Reeler* mice, which lack Reelin and the Reelin receptor [apolipoprotein E receptor 2 (ApoER2)/very low density lipoprotein receptor (VLDLR)], there are similar hippocampal histological abnormalities as those that occurred here in MIA offspring. Notably, these abnormalities include the aberrant migration of granular cells, as well as deregulated projections of mossy fibers (Drakew et al., 2002). Given that Reelin-positive interneurons are abundant near the dentate granular cells of the hippocampus, secreted Reelin may activate ApoER2/VLDLR on granular cells in the DG, which could lead to the formation of mossy fibers sprouts from granular cells. Thus, decreased numbers of Reelin-positive cells may play a causative role in the down-regulation of synaptoporin-immunostained area in MIA offspring. This possibility is supported by our present results, in which Reelin injection rescued synaptoporin deficit in the hippocampi of MIA offspring (Figures 4B,C).

The downstream Reelin signaling pathway should be considered when theorizing about the molecular mechanisms underlying its possible therapeutic effects. Reelin receptors (ApoER2 and VLDLR) can phosphorylate Disabled-1 (Dab1), which is a Reelin adaptor protein (Herz and Chen, 2006). This pathway downstream of Reelin activates phosphatidylinositol-3-kinase (PI3K) and subsequently stabilizes the cytoskeleton, contributing to cell migration. Further, Reelin activates the N-methyl-D-aspartate (NMDA) receptor, and in turn the cAMP response element binding protein (CREB), leading to enhanced synaptic plasticity and development (Herz and Chen, 2006).

Indeed, mice either with Reelin supplementation into the brain (Rogers et al., 2011) or with overexpression of Reelin (Pujadas et al., 2010) exhibit enhancement of cognition and long-term potentiation (LTP). In addition, single nucleotide polymorphisms (SNPs) in the *APOER2*, *VLDLR*, and *DAB1* genes are associated with cognitive impairments in patients with schizophrenia (Verbrugghe et al., 2012). We have further identified a novel exonic deletion of *RELN* in a patient with schizophrenia (Sobue et al., 2018), and mice with this *RELN* mutation exhibit schizophrenia-like behaviors and histological abnormalities (Sobue et al., 2018). While these results suggest that signaling pathways downstream of Reelin may contribute to its therapeutic effects (Herz and Chen, 2006; Rogers et al., 2011; Verbrugghe et al., 2012; Hethorn et al., 2015), the molecular mechanisms by which Reelin recovers brain dysfunction in MIA remain unknown. Further study in preclinical models of neurodevelopmental disorders is required to clarify the effects of Reelin downstream from its lipoprotein receptors.

Regarding the microinjection of recombinant Reelin, the present study has a limitation not to clarify how far injected recombinant Reelin spread. Instead of recombinant Reelin, we have investigated the diffusion of a dye (methylene blue) injected into the hippocampus (Supplementary Figure S1), in which it looks confined to the hippocampal DG, suggesting that recombinant Reelin may stay around the DG following the injection. In addition, we have already found that injection of recombinant Reelin into the hippocampus had no effect on the hippocampal Reelin protein levels in mice at the end of behavioral analyses, 2 weeks after the injection (data not shown), which is consistent with a previous report to indicate that recombinant Reelin injected into the brain is maintained only in a few hours, and subsequently degraded (Rogers et al., Learn Mem 2011). On the other hand, some studies have demonstrated that injection of recombinant Reelin into the brain increases the number of spine and enhances synaptic plasticity even 5–10 days after the injection, which is corelated with the amelioration in cognitive function (Rogers et al., 2011, 2013; Hethorn et al., 2015; Ishii et al., 2015). These previous findings raise a possibility that recombinant Reelin injection into the hippocampus activates the Reelin receptors (i.e., ApoER and VLDLR) around the hippocampal DG, leading to the enhancement of synapse formation/function as well as cognition. These effects may be supposedly maintained in a certain period of time even though injected recombinant Reelin had been degraded in a few hours after the injection. Further study is required to elucidate the mechanism underlying the long-lasting effect of injected Reelin on synapse and cognition.

Prenatal stressors including MIA and physical restraint decrease hippocampal Reelin expression in offspring (Meyer et al., 2006; Palacios-Garcia et al., 2015). DNA hypermethylation of the *RELN* promoter also contributes to Reelin down-regulation (Palacios-Garcia et al., 2015). Clinical evidence supports increased methyl donor S-adenosylmethionine in the brains of patients with schizophrenia (Guidotti et al., 2007), and an association between hypermethylation of the *RELN* promoter (Grayson et al., 2006) and down-regulation of the corresponding protein (Reelin) in the brains of these patients (Guidotti et al., 2016). This suggests that hypermethylation of the *RELN* promoter in both animal models and in patients with neurodevelopmental disorders epigenetically suppresses Reelin expression in the hippocampus, leading to cognitive and synaptic disturbances. The release of this suppression from hypermethylation of the *RELN* promoter may thus decrease down-regulation of Reelin protein expression in the brain, which could serve as an alternative therapeutic strategy to direct Reelin injection in the brain.

Another therapeutic approach to the treatment of Reelin dysregulation in schizophrenia may be the inhibition of the enzyme that degrades Reelin. A recent report demonstrated that A Disintegrin and Metalloproteinase with Thrombospondin motifs 3 (ADAMTS-3) is the major enzyme involved in Reelin cleavage and inactivation (Ogino et al., 2017). Previous reports have already demonstrated that mice with truncated inactive Reelin exhibit schizophrenia-like behaviors such as hyperactivity, social withdrawal, and memory deficits (Sakai et al., 2016; Sobue et al., 2018). Inhibition of ADAMTS-3 may therefore reduce the amount of inactive Reelin and help to improve the symptoms seen in neurodevelopmental disorders. Futures studies should examine these impacts preclinically for potential translation to humans.

In the present study, we have demonstrated that MIA enhanced the locomotor -activity (Supplementary Figure S2) and anxiety-like behavior (Figure 3) in offspring, supposedly associating with positive and negative symptoms, respectively. Further, offspring with MIA exhibited the impairment of recognition memory (Figure 3), reflecting with cognitive symptom in schizophrenia (McGuire et al., 2013). Meanwhile, MIA had no effect on the social behaviors and sensorimotor gating in social interaction and PPI tests, respectively [social interaction: *p* = 0.26 (Mann-Whitney *U* test); PPI: F_PolyI:C (1,27)_ = 0.072, *p* = 0.79 (two-way ANOVA)]. These results suggest that MIA model prepared under our experimental condition may show only a part of behavioral abnormalities as observed in schizophrenia, which partially achieve an adequate level of behavioral validity as previously reported (Jones et al., 2011). On the other hand, the most studied and reproducible cognitive impairments in schizophrenics is working memory deficit (Elvevåg and Goldberg, 2000). In rodents, working memory can be measured in various tasks such as the eight-arm radial maze test and delayed non-matching to sample position operant conditioning task (Dudchenko, 2004). For the further verification of validity as an animal model of schizophrenia, memory function in the present MIA model should be investigated in future studies by using such behavioral cognitive tests.

## Data Availability Statement

All datasets presented in this study are included in the article/Supplementary Material.

## Ethics Statement

The animal study was reviewed and approved by the Animal Ethics Board of Meijo University.

## Author Contributions

DI, TaN, ToN, KY, and MHi designed the experiments, analyzed the data, and wrote the manuscript. GN, NK, and RT performed the experiments. MS, TK, and MHa helped with purification of recombinant Reelin protein. All authors reviewed, edited, and approved the final manuscript.

## Conflict of Interest

The authors declare that the research was conducted in the absence of any commercial or financial relationships that could be construed as a potential conflict of interest.
