# Supplementary figures and images for: Reelin Supplementation Into the Hippocampus Rescues Abnormal Behavior in a Mouse Model of Neurodevelopmental Disorders
Source: Front Cell Neurosci. 2020 Sep 2;14:285. doi: 10.3389/fncel.2020.00285 (PMC7492784; doi:10.3389/fncel.2020.00285)

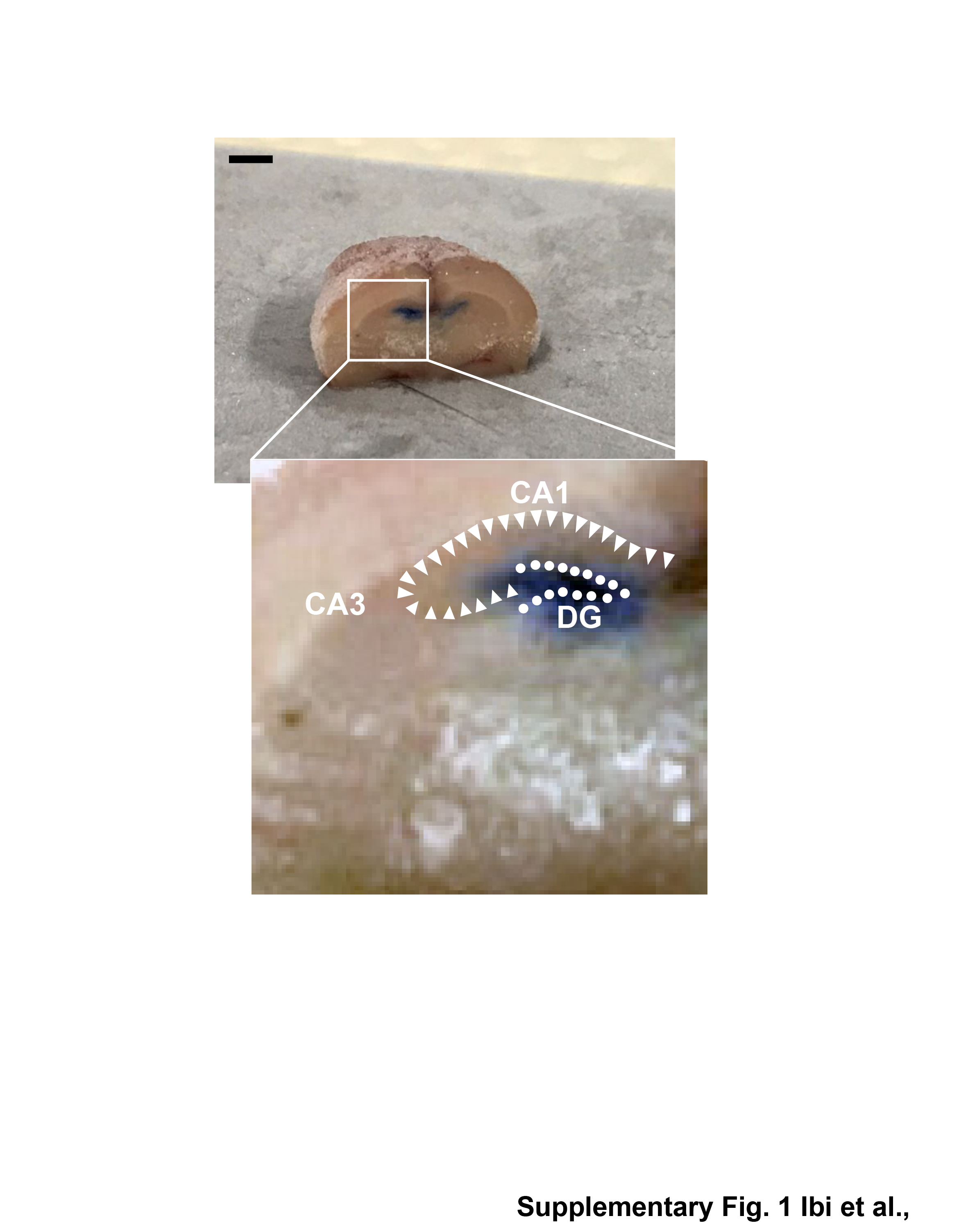

Supplement: Supplementary file 1 [file Image_1.TIF]

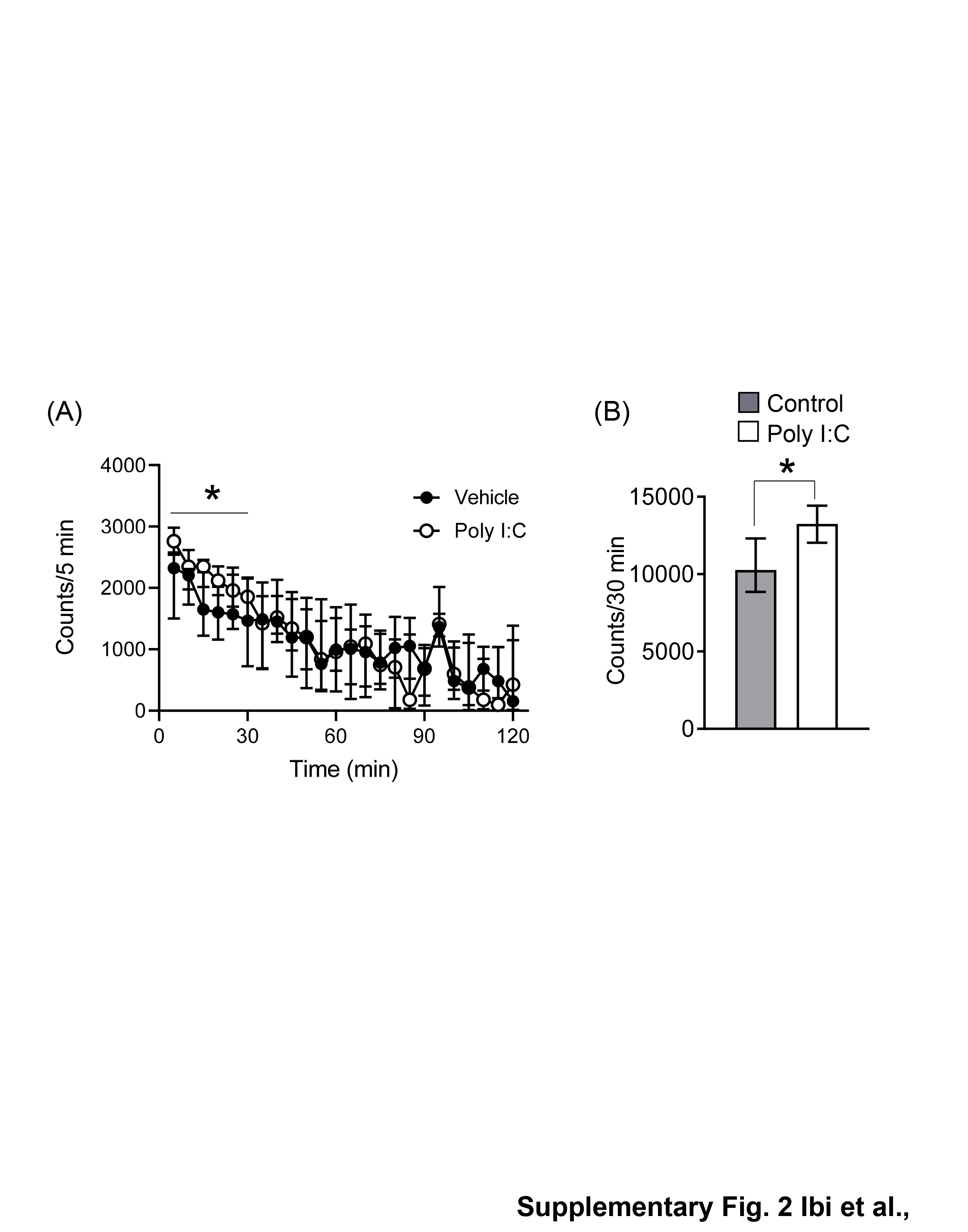

Supplement: Supplementary file 2 [file Image_2.TIF]
