# Supplementary material for: Reelin Supplementation Into the Hippocampus Rescues Abnormal Behavior in a Mouse Model of Neurodevelopmental Disorders
Source: Front Cell Neurosci. 2020 Sep 2;14:285. doi: 10.3389/fncel.2020.00285 (PMC7492784; doi:10.3389/fncel.2020.00285)
Supplement: Supplementary file 3 [file Table_1.DOCX]

**Figure legends**

**Supplementary Figure 1. Verification the accuracy of hippocampal injection by** **methylene blue.**

Coronal section image after methylene blue injection into the hippocampus. Methylene blue injected stays around hippocampal DG. Scale bar: 2 mm.

**Supplementary Figure 2. Spontaneous locomotor activity in offspring of dams exposed to immune activation.**

Locomotor activity shown in 5-min blocks for 120 min (A) and quantified from t=5 to t =30 min (B). Values represent the median and interquartile range (n=10-12 males). Bonferroni's test (A); Mann-Whitney U test (B). **p*<0.05 vs. vehicle-treated control group.
